# Noradrenergic correlates of chronic cocaine craving: neuromelanin and functional brain imaging

**DOI:** 10.1038/s41386-020-00937-9

**Published:** 2021-01-06

**Authors:** Wuyi Wang, Simon Zhornitsky, Sheng Zhang, Chiang-shan R. Li

**Affiliations:** 1 Department of Psychiatry, Yale University School of Medicine, New Haven, CT 06520 USA; 2 Department of Neuroscience, Yale University School of Medicine, New Haven, CT 06520 USA; 3 Interdepartmental Neuroscience Program, Yale University, New Haven, CT 06520 USA

**Keywords:** Predictive markers, Motivation

## Abstract

Preclinical studies have implicated noradrenergic (NA) dysfunction in cocaine addiction. In particular, the NA system plays a central role in motivated behavior and may partake in the regulation of craving and drug use. Yet, human studies of the NA system are scarce, likely hampered by the difficulty in precisely localizing the locus coeruleus (LC). Here, we used neuromelanin imaging to localize the LC and quantified LC neuromelanin signal (NMS) intensity in 44 current cocaine users (CU; 37 men) and 59 nondrug users (NU; 44 men). We also employed fMRI to investigate cue-induced regional responses and LC functional connectivities, as quantified by generalized psychophysiological interaction (gPPI), in CU. Imaging data were processed by published routines and the findings were evaluated with a corrected threshold. We examined how these neural measures were associated with chronic cocaine craving, as assessed by the Cocaine Craving Questionnaire (CCQ). Compared to NU, CU demonstrated higher LC NMS for all probabilistic thresholds defined of 50–90% of the peak. In contrast, NMS of the ventral tegmental area/substantia nigra (VTA/SN) did not show significant group differences. Drug as compared to neutral cues elicited higher activations of many cortical and subcortical regions, none of which were significantly correlated with CCQ score. Drug vs. neutral cues also elicited “deactivation” of bilateral parahippocampal gyri (PHG) and PHG gPPI with a wide array of cortical and subcortical regions, including the ventral striatum and, with small volume correction, the LC. Less deactivation of the PHG (*r* = 0.40, *p* = 0.008) and higher PHG-LC gPPI (*r* = 0.44, *p* = 0.003) were positively correlated with the CCQ score. In contrast, PHG-VTA/SN connectivity did not correlate with the CCQ score. Together, chronic cocaine exposure may induce higher NMS intensity, suggesting neurotoxic effects on the LC. The correlation of cue-elicited PHG LC connectivity with CCQ score suggests a noradrenergic correlate of chronic cocaine craving. Potentially compensating for memory functions as in neurodegenerative conditions, cue-elicited PHG LC circuit connectivity plays an ill-adaptive role in supporting cocaine craving.

## Introduction

### Noradrenergic dysfunction in cocaine addiction

Although not as thoroughly investigated as dopamine (DA), norepinephrine (NE) has consistently been implicated in cocaine misuse [1–5]. For instance, in squirrel monkeys trained to stability and then extinguished for self-administration of cocaine, priming with NE transporter (NET) inhibitors reinstates drug seeking [3, 5]. In rodents too, noradrenergic (NA) agents have a robust effect on the reinstatement of stimulant seeking [5]. In humans NET genetic polymorphism modulates subjective mood responses to d-amphetamine in healthy individuals [6]. The polymorphisms are located at the transcription factor binding sites and thus likely to regulate the expression of NET gene. Relatively little is known about the effects of prolonged stimulant use on NA neurotransmission. Postmortem and animal studies have provided evidence in support of upregulation of NET after chronic cocaine exposure [7–9]. A more recent in-vivo study similarly demonstrated that NET is upregulated in humans addicted to cocaine [10]. Together, both clinical and preclinical studies support the role of NA signaling in the shaping and maintenance of cocaine addiction.

### Locus coeruleus, motivated behavior, and drug craving

Both DA and NA systems are involved in motivating goal-directed behavior [11–14]. DA is best known for signaling unexpected and predicting upcoming reward [15]. More recent research has explored the role of DA in signaling aversive events [16, 17], broadening the scope of DA responses to saliency. Research of the NA system, on the other hand, has focused on its function in supporting arousal, attention, and stress-related behavior [18–21]. In particular, studies have accumulated to suggest the contribution of the NA circuits to effort and motivated behavior [22]. For instance, administration of clonidine, an α-2 NE receptor agonist that reduces central NE levels, decreased choice variability and force production in monkeys engaged in a sequential cost/benefit decision task [23]. Stimulant abuse may compromise the arousal-promoting function of the NA circuits and lead to dysmotivation syndrome [24]. In monkeys performing a task that involved reward/effort trade-off, NA neurons of the locus coeruleus (LC) increased in activity with both pupil dilation and effort production during reward-seeking behavior [25], suggesting a specific role of the LC in energizing behavior to challenges [26]. It has been suggested that combining molecular and/or neuromelanin imaging and functional magnetic resonance imaging (fMRI) is central to understanding the catecholaminergic processes of motivated behaviors in health and illness [27].

Drug seeking reflects a motivated behavior, and individuals with heavier use tend to demonstrate higher craving and more compulsive drug seeking [28–30]. Many imaging studies have employed cue-reactivity paradigms to investigate the neural processes of craving [31–38] and how the neural processes predict drug use and treatment outcome [39–42]. Drug craving is associated with biased attention to drug-related stimuli and physiological arousal, as supported by the NA circuit, including the midbrain nucleus LC [18]. Electrophysiological recordings show that the LC neurons respond to approach behavior, as during timed go trials in a go/no-go task [43], and most vigorously to complex arousing stimuli such as a preferred food [44]. It is thus highly likely that regional responses to drug cues interact with the LC to support craving and drug use.

### Neurotoxic effects of chronic cocaine exposure

Chronic use of stimulants is known to be associated with neurotoxicity, potentially by way of excitotoxicity, mitochondrial and endoplasmic reticulum dysfunction, oxidative stress, and neuroinflammation [45–47]. Many of these neurotoxic processes have also been implicated in neurodegenerative changes as in Parkinson’s and Alzheimer’s disease, with studies suggesting earliest manifestation in the NA system that can be quantified by neuromelanin imaging [48]. Neuromelanin is identified in monoamines-containing neurons and formed by polymerization of 4,5-dihydroxyindole monomers [49] via enzymatic processes that involve the monoamine oxidase [50]. Neuromelanin chelates metals and protects against oxidative stress [51, 52]. Importantly, the cellular stress is evident during aging in neurotypical populations, with the LC neuromelanin contrast increasing until the sixth decade of life, when the compensatory process starts to fall apart, and decreasing afterwards [53, 54]. Thus, NMS of the LC does not simply suggest neurotoxicity but also reflect a protective mechanism for functional compensation, as observed during healthy aging [45]. With transcranial sonography an earlier study demonstrated higher echogenicity of the substantia nigra in stimulant users, as compared to controls and cannabis users, suggesting iron accumulation and microglia activation [55]. However, no studies to date have directly investigated neuromelanin signals (NMS) of the monoaminergic nuclei in cocaine users.

### The present study

Here, we employed neuromelanin imaging to localize the LC and quantify its signal intensity as well as fMRI to query whole-brain responses and how these regional activities interact with the LC during exposure to drug vs. neutral cues in cocaine users (CU). We hypothesized higher NMS in cocaine users as compared to nonusers and higher regional cue-elicited connectivity with the LC in correlation with chronic cocaine craving in CU.

## Participants and methods

### Subjects and assessments

All participants signed an informed consent according to a protocol approved by the Human Investigation Committee at Yale University. Forty-four current cocaine users (CU; mean ± SD: 46.5 ± 7.2 years of age; 7 women) and 59 nonusers (NU; 43.7 ± 9.8 years, 15 women) were recruited from the greater New Have area of Connecticut. CU met Diagnostic and Statistical Manual of Mental Disorders IV (DSM-IV) criteria for cocaine dependence [56], and tested positive for cocaine in urine toxicology. All were required to be physically healthy with no major medical illnesses, other Axis I disorders (except nicotine use disorders), current use of prescription medications, or history of head injury or neurological illness. CU who reported current use of other illicit substances or tested positive for methamphetamine, opioids, marijuana, barbiturates, or benzodiazepines were not invited to participate. Participants stayed at a locked inpatient unit at the Connecticut Mental Health Center and participated in the MR scan in 1–2 weeks. Participants were evaluated with the Cocaine Craving Questionnaire [57], Alcohol Use Disorders Identification Test [58], and the Fagerström Test for Nicotine Dependence [59]. Table 1 summarizes the demographic and clinical characteristics of the participants.

**Table 1:** Demographics and clinical measures of CU and NU participants.

	CU (*n* = 44)	NU (*n* = 59)	*t*	*p*
Age (years)	46.5 ± 7.2	43.7 ± 9.8	1.251	0.214
Gender (M/F)	37/7	44/15	1.358	0.244ª
Years of cocaine use	17.6 ± 9.9	N/A	N/A	N/A
Days of cocaine use, prior month	19.2 ± 8.5	N/A	N/A	N/A
Total use (gm), prior month	30 ± 39	N/A	N/A	N/A
CCQ score	40.0 ± 16.2	N/A	N/A	N/A
AUDIT score	4.8 ± 5.7	3.7 ± 4.3	0.929	0.355
FTND score	3.6 ± 2.8	0.3 ± 1.2	8.283	<0.001

All values are mean ± S.D.

*P* values are based on independent-sample *t* test, except for gender compositiona (*x*^2^ test).

*CCQ* Cocaine Craving Questionnaire, *AUDIT* Alcohol Use Disorder Identification Test, *FTND* Fagerström Test for Nicotine Dependence.

### Cue-reactivity task for fMRI

We employed a cue-reactivity task for fMRI as in a recent work [60]. Participants viewed cocaine-related or neutral pictures in alternating blocks. Briefly, a cross was used to engage attention at the beginning of each block. After 2 s, six pictures displaying cocaine-related cues (cocaine block) or neutral visual scenes (neutral block) were shown for 6 s each. Participants were asked to view the pictures and ponder how they might relate to the scenes. The pictures were collected from the internet and independently reviewed by two investigators. Cocaine pictures included individuals or a group of people using cocaine or images of cocaine. Neutral pictures comprised natural sceneries. Each block lasted about 45 s. A total of six cocaine and six neutral blocks took ~9 m to complete in each “run”. Each CU completed two runs of the task.

### Imaging protocol and data preprocessing

Imaging data were obtained on a 3-Tesla Siemens Prisma scanner with multiband sequence. T1-weighted spin echo sagittal anatomical images were acquired for slice localization. A straight axial T1 FLASH structural image was obtained with TR = 440 ms, TE = 2.61 ms, bandwidth = 500 Hz/pixel, field of view = 220 × 220 mm, matrix = 256 × 256, 47 slices with 2.5 mm thickness and 1 mm gap. Four neuromelanin-sensitive-weighted MRI scans covering the LC and VTA/SNc were collected using a T1-weighted FSE sequence (straight axial, TR = 753 ms, TE = 12 ms, bandwidth = 222 Hz/pixel, flip angle = 120°, field of view = 220 × 220 mm, matrix = 512 × 512, 15 slices with 2.5 mm thickness and 1 mm gap, in-plane resolution = 0.429 × 0.429 mm^2^). The slices’ center was copied from the T1 FLASH image and moved down a number of slices, as adjusted for individual subjects to ensure LC coverage. This individual-specific number allowed us to insert the TSE scan to T1 FLASH template for normalization (see below). Anatomical 3D MPRAGE image were next obtained with spin echo imaging with TR = 2400 ms, TE = 1.18 ms, bandwidth = 610 Hz/pixel, field of view = 256 × 256 mm, matrix = 256 × 256, 208 slices at 1 mm and no gap. Functional, blood oxygen level-dependent (BOLD) signals were acquired with a single-shot gradient echo echoplanar imaging sequence. Seventy-five straight axial slices covering the whole brain were acquired with TR = 1000 ms, TE = 30 ms, bandwidth = 1894 Hz/pixel, flip angle = 55°, field of view = 220 × 220 mm, matrix = 110 × 110, 75 slices with 2 mm thickness and no gap.

We analyzed the imaging data with Statistical Parametric Mapping or SPM12. Because the FSE scan included only part of the brain, we used the resampled T1 FLASH image for normalization and applied the normalization parameters on the LC image. The T1 FLASH image was first resampled to match the in-plane resolution of the LC image (0.429 × 0.429 mm^2^). The 15 slices of the LC image were inserted into a 47-slice template in the same dimension with the resampled T1 FLASH image, generating a new LC image. In this way, the normalization parameters applied to resampled T1 FLASH image could be applied to the new LC image too. The MPRAGE image was co-registered with the resampled T1 FLASH image and then segmented for normalization with affine registration followed by nonlinear transformation [61–63]. The normalization parameters were then applied to the corresponding new LC images with a voxel size of 0.5 × 0.5 × 2 mm^3^.

In preprocessing of the BOLD signals, we aligned (motion-corrected) and corrected for slice timing of images of each individual subject, and constructed a mean functional image volume for each subject per run from the realigned volumes. We co-registered these mean images with the MPRAGE image and segmented the images for normalization with affine registration and nonlinear transformation. We applied the normalization parameters as determined for the structure volume to the corresponding functional images for each subject. The resampled voxel size was 3 × 3 × 3 mm^3^.

### Controlling for physiological noise

The LC is adjacent to the fourth ventricle, and the BOLD signals in this region can potentially be affected by physiological noise. We used the DRIFTER SPM toolbox (http://becs.aalto.fi/en/research/bayes/drifter/) to remove the respiration and cardiac noises on the basis of a Bayesian model [64], as in our earlier work [65]. Briefly, the frequency trajectories of the physiological signals were first estimated by the interacting multiple models filter algorithm. The number of periodics to estimate was set as 1 to remove both respiration and cardiac noises. In a state-space model in combination of a Kalman filter and Rauch-Tung-Striebel smoother, BOLD time courses were separated into cleaned activation-related signal, physiological noise, and white noise.

### Imaging data modeling

In modeling of the functional data, we distinguished cocaine and neutral cue blocks for each individual subject using a general linear model (GLM) that included the realignment parameters in all six dimensions. We corrected for serial autocorrelation caused by aliased cardiovascular and respiratory effects by a first-degree autoregressive model. We constructed for individual subjects a contrast of cocaine vs. neutral blocks to evaluate regional activities that differentiated viewing of cocaine and neutral pictures. The *con* (difference in *β*) images were used for group-level, random-effects analyses. In region of interest (ROI) analysis, we used MarsBaR (http://marsbar.sourceforge.net/) to derive for each individual subject the *β* contrast or “drug − neutral” activity for the ROIs. We showed all voxel activations in the Montreal Neurological Institute (MNI) coordinates and identified the brain regions by referring to an atlas [66].

### Psychophysiological interaction (PPI)

We examined PPI with the LC during cue exposure (drug vs. neutral). PPI describes functional connectivity between brain regions contingent on a psychological context [67]. We used a generalized form of PPI (gPPI), where the inclusion of task regressors reduces the likelihood that the functional connectivity estimates are driven simply by co-activation [68]. The extracted mean time series of the BOLD signals were temporally filtered, mean corrected, and de-convolved to generate the time series of the neural signal for the LC mask for each individual subject to compose the physiological variable. These time series were then multiplied by the onset times of the blocks of interest separately, and re-convolved with the canonical HRF to obtain the interaction term or PPI variable. Finally, the blocks of interest, the physiological variable, and PPI variables were entered as regressors in a whole-brain GLM. GPPI analysis was performed for each individual subject, and the resulting contrast images were used in random-effects analyses. We tested specifically whether regional responses to drug (vs. neutral) cues interact with the LC and/or VTA/SNc, as defined by neuromelanin imaging (see below) and whether these gPPIs were correlated with CCQ scores.

### Neuromelanin signal intensity

We computed for individual subjects the NMS of the LC and, as a contrast, the ventral tegmental area/substantia nigra, pars compacta (VTA/SNc), with the superior cerebellar peduncle (SCP) as a control region [69]: (LC − SCP)/(LC + SCP) and (VTA/SNc − SCP)/(VTA/SNc + SCP), respectively. We used a probabilistic template of the LC derived earlier [70] that represents the extent of peak LC signal distribution with a volume of 93 mm^3^. The template of the VTA/SNc was derived from structural MRIs [71] with a volume of 1106 mm^3^. We obtained the SCP template from the AAL atlas [72].

First, we verified for all subjects that the voxel with peak NMS lay within the LC and VTA/SNc template. Second, rather than computing the mean signal intensity of the masks, we considered voxels within an area that extended beyond the LC and VTA/SNc templates, considering that individual participants may vary in how LC and VTA/SNc voxels were distributed. This extended area comprised the voxels of the templates and three and one additional layers of voxels for the LC and VTA/SNc (797 and 2276 mm^3^), respectively. Finally, we computed each for the LC and VTA/SNc the average NMS for voxels with intensities exceeding a threshold each of 50–90% of the peak, using the afore-described formula. The masks of LC and VTA/SNc as identified served as the ROIs for gPPI analyses. Specifically, we tested the correlations of cue-elicited regional gPPI with the LC and VTA/SNc defined across multiple thresholds to ensure that the results were robust.

## Results

### Clinical assessments

As shown in Table 1, CU and NU did not differ in age, sex composition, or AUDIT score. CU showed significantly higher FTND score than NU. In all data analyses, we included age, sex, AUDIT and FTND scores as covariates. In CU, the CCQ score was positively correlated with days of cocaine use in the prior month (*p* = 0.010, *r* = 0.41) as well as total cocaine use in the prior month (*p* = 0.034, *r* = 0.34). In subsequent analyses, we focused on the CCQ score as an index of chronic craving, as in our previous studies [60, 73, 74].

### Neuromelanin imaging of the LC and VTA/SNc

**Fig. 1:**
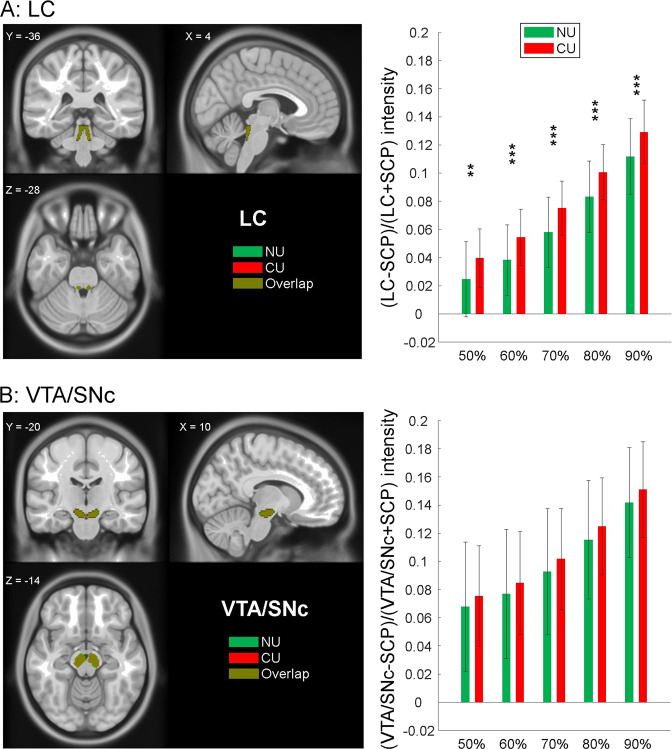
NMS of the LC and VTA/SNc in CU and NU. **A** LC NMS intensities were significantly different between CU and NU across all thresholds. **B** VTA/SNc NMS intensities were not significantly different between CU and NU across the same thresholds. Histograms show mean ± SD of the NMS for CU and NU at each threshold. **p < 0.01; ***p < 0.001; two-sample two-tail *t*-test with age, sex, AUDIT and FTND scores as covariates.

Relative to NU, CU showed higher NMS in the LC. Figure 1A shows the intensity values for different extents of the LC region, each covering voxels with ≥50–90% of the peak intensity. The NMS of the VTA/SNc did not show significant differences between CU and NU (Fig. 1B). The NMS of the LC was not correlated with years of cocaine use (*r* = 0.04, *p* = 0.788) or with the estimated life-time amount of cocaine use (*r* = 0.01, *p* = 0.969).

### Cue-elicited regional activations in CU

**Fig. 2:**
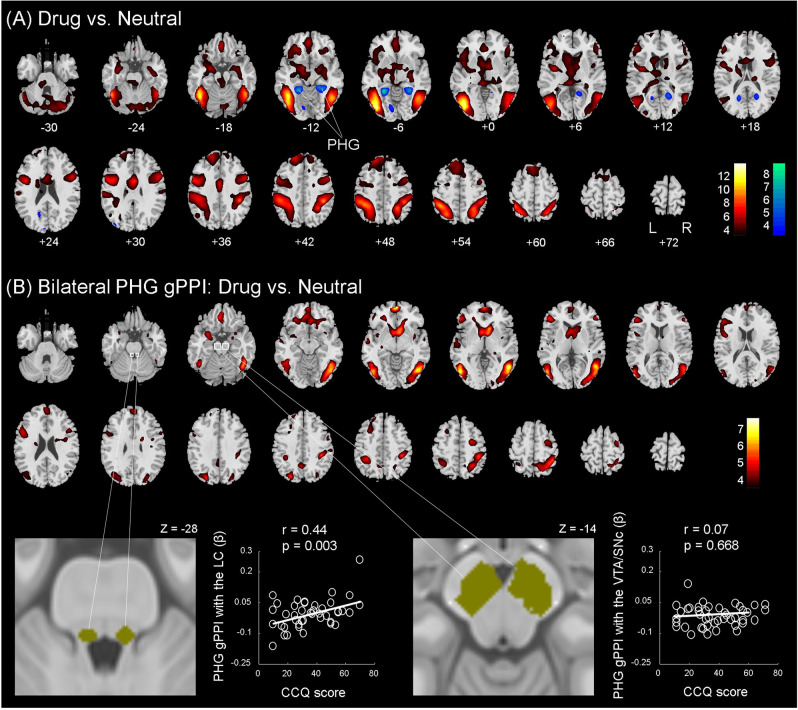
Cue-induced regional activations and functional connectivity. **A** One-sample *t* test of drug vs. neutral cues in CU. Voxels showing higher activation during drug vs. neutral and neutral vs. drug are shown in warm and cool colors, respectively. Voxel *p* < 0.001, uncorrected. Clusters that met cluster *p* < 0.05, FWE-corrected are listed in Table 2. **B** (upper panel) Cue-induced functional connectivity (gPPI) with bilateral PHG in CU: one-sample *t* test of drug vs. neutral cues. Voxels showing higher gPPI during drug vs. neutral are shown in warm colors. Voxel *p* < 0.001, uncorrected. Clusters that met cluster *p* < 0.05, FWE-corrected are listed in Supplementary Table S1. No clusters showed higher gPPI in response to neutral vs. drug cues. **B** (lower panel) LC and VTA/SNc masks and linear regression of LC and VTA/SNc gPPI with PHG vs. CCQ scores in CU.

Figure 2A shows the one-sample *t* test results of drug vs. neutral cues in CU. Bilateral lateral occipital, temporal, and fronto-parietal cortices, cerebellum, and medial frontal cortex showed higher activations to drug vs. neutral cues. Conversely, two clusters in bilateral parahippocampal gyrus (PHG) showed lower activations to drug vs. neutral cues. These clusters are summarized in Table 2.

**Table 2:** Cue-induced activations: one-sample *t* test of drug vs. neutral in CU.

Regions	Cluster	Voxel	MNI coordinates (mm)		
	Size (voxels)	*Z* value	*X*	*Y*	*Z*
Activation: drug > neutral cue*					
L inferior occipital G	905	Inf	−42	−76	−2
R inferior temporal G	634	7.45	45	−61	−8
R supramarginal G	537	7.18	42	−37	40
L superior parietal lobule	788	7.18	−39	−46	55
Middle cingulate cortex	88	6.38	3	2	31
L precentral G	211	6.03	−48	2	34
L cerebellum	109	5.91	−21	−73	−50
R cerebellum	202	5.87	12	−76	−44
L superior frontal G	173	5.73	−18	47	43
R precentral G	157	5.64	51	11	25
Ventromedial PFC	58	5.61	0	35	−23
L anterior insula	30	5.47	−36	−4	7
L amygdala	51	5.16	−33	−10	−23
L thalamus	12	5.04	−3	−31	4
L caudate	24	4.99	−27	35	−14
Activation: drug < neutral cue					
L parahippocampal G	115	−6.62	−27	−46	−8
R parahippocampal G	118	−5.76	27	−43	−8

An asterisk indicates clusters surviving peak voxel *p* < 0.05 FWE corrected, with the cluster size shown for voxels meeting this threshold; L and R parahippocampal clusters also showed a peak voxel *Z* value that met voxel *p* < 0.05 FWE corrected; however, the cluster sizes are shown for voxel *p* < 0.001, uncorrected—thus, these clusters correspond exactly in size to those shown in Fig. 2A.

*L* left, *R* right, *G* gyrus, *PFC* prefrontal cortex.

We extracted the *β* contrasts (drug − neutral) of all identified clusters and observed with linear regressions that only bilateral PHG (left PHG, in particular) demonstrated cue-elicited activities (drug − neutral) in positive correlation with CCQ score, with age, sex, AUDIT and FTND scores as covariates (*r* = 0.38, *p* = 0.017, bilateral PHG; *r* = 0.46, *p* = 0.002, left PHG). That is, the PHG showed “deactivations” to drug vs. neutral cues and less deactivations were associated with higher chronic cocaine craving. None of the other clusters demonstrated cue activities in correlation with CCQ scores (all *p*’s > 0.141). The statistics of Pearson regressions are summarized in Table 3.

**Table 3:** Correlation of regional cue-related activity with the CCQ score in CU.

	CCQ score	
	*P*	*R*
All positive clusters combined	0.212	0.19
L inferior occipital/fusiform/inferior temporal G	0.138	0.23
Supramarginal G/superior parietal lobule	0.982	0.00
Superior parietal lobule/supramarginal G	0.429	0.12
Superior frontal G	0.253	0.18
Precentral/inferior frontal G	0.826	0.03
All negative clusters combined	0.008*	0.40
L parahippocampal G	0.002*	0.46
R parahippocampal G	0.053*	0.30

Positive clusters: drug > neutral; negative clusters: neutral > drug.

*L* left, *R* right, *G* gyrus.

*p < 0.05.

### Cue-elicited functional connectivity

Bilateral PHG showed cue-elicited activity in positive correlation with the CCQ score. Thus, we conducted a functional connectivity analysis to examine the generalized psychophysiological interaction (gPPI) of the bilateral PHG during exposure to drug vs. neutral cues in CU. We performed voxelwise analyses and the results of one-sample *t* test of gPPI are shown in Fig. 2B. The clusters meeting cluster threshold *p* < 0.05 FWE-corrected are summarized in Supplementary Table S1.

For individual clusters showing significant gPPI with bilateral PHG, we examined the correlation of the gPPI *β* values with the CCQ score and none showed a significant correlation (all *p*’s > 0.041, uncorrected).

To test our hypotheses of functional connectivity with midbrain nuclei, we computed the *β* contrast of gPPI for the LC mask (≥50% of peak NMS) and observed that bilateral PHG gPPI with the LC was significantly correlated with the CCQ score (*r* = 0.44, *p* = 0.003), with age, sex, AUDIT and FTND scores as covariates (Fig. 2B, lower left panel). The correlations were also significant for LC mask defined of 60% (*p* = 0.005), 70% (*p* = 0.026), and 80% (*p* = 0.035). For the mask defined of >90% intensity of the peak (comprising only 7.7 ± 9.6 mm^3^), the correlation was marginally significant (*p* = 0.058). Notably, with only the left PHG as seed, the findings remained similar: left PHG gPPI with the LC was significantly correlated with the CCQ score, with age, sex, AUDIT and FTND scores as covariates, for the LC mask defined of 50% (*r* = 0.37, *p* = 0.017), 60% (*p* = 0.022), and 70% (*p* = 0.049) intensity of the peak. In contrast, the gPPIs of the VTA/SNc as defined of 50% (*p* = 0.668, Fig. 2B, lower right panel), 60% (*p* = 0.687), 70% (*p* = 0.816), 80% (*p* = 0.586) and 90% (*p* = 0.397) of the peak intensity were not significantly correlated with the CCQ score. Thus, chronic cocaine craving was specifically associated with higher PHG gPPI with the LC.

## Discussion

The current results are the first to show altered NMS intensity of the LC in CU. Further, with neuromelanin imaging to localize the LC in individual CU, we demonstrate cue-elicited LC functional connectivity in relation to chronic cocaine craving. These findings together provide evidence in humans for NA dysfunction in cocaine addiction. The VTA/SNc also showed higher NMS in CU than in NU, but the differences did not reach statistical significance. VTA/SNc connectivity with the PHG was not correlated with chronic cocaine craving. These findings together suggest an outsized impact of chronic cocaine exposure on the NA system and its more prominent role in supporting chronic cocaine craving.

### Cocaine-induced neurotoxic effects on the LC

Previous investigations of the neurotoxic effects of stimulants, including cocaine, have focused on the dopaminergic system [46]. Here, we demonstrated significantly higher NMS in the LC in CU, as compared to NU, suggesting neurotoxic effects of cocaine on the NA system. As described earlier, the levels of NET are upregulated, indicating reduction of NA signaling, both in rhesus monkeys and humans chronically exposed to cocaine [8, 10]. A previous study reported a strong positive correlation between NMS intensity and NET levels in the LC of postmortem human brain [75]. Another study showed a significant inverse relationship between the number of remaining LC neurons and NE metabolism in the frontal cortex and LC, suggesting that remaining LC neurons are activated to compensate for decreased cerebral NE levels, in individuals with Alzheimer’s disease. This compensatory process, as reflected in LC connectivity with the hippocampus and PHG, may support memory functions in these neurodegenerative conditions [76–79].

Both in vivo and in vitro studies have reported that prenatal cocaine exposure inhibits neurite formation and induces LC neuronal damage and apoptosis [80, 81]. In an in-vitro study cocaine exposure preferentially induced LC cell apoptosis by disrupting membrane skeleton integrity and DNA repair, without significant effects on the survival of substantia nigra neurons [82]. In accord, we observed here significant higher LC but indistinguishable VTA/SNc NMS intensity in CU as compared to NU, suggesting that the LC neurons may be more vulnerable to cocaine exposure. On the other hand, this finding does not rule out neurotoxicity on dopaminergic neurons, which could potentially be demonstrated with a larger sample size. The findings together suggest that NMS intensity of the LC, as can be quantified by turbo-spin echo imaging, may represent a marker of the neurotoxic effects of cocaine on the NA system. On the other hand, it is noted that LC NMS intensity did not correlate with years of cocaine use or estimated life-time cocaine use in CU. It is possible that life-time cocaine consumption is difficult to quantify in chronic users, many with varying severity of cocaine use, including a hiatus in use, throughout their lifetime. The monoamingeric nuclei are vulnerable to other environmental toxicants, including pesticides and heavy metals. Further, as discussed earlier, the NMS does not simply suggest neurotoxicity but also reflect a neural protective mechanism.

### Cue-related regional responses in cocaine users

Drug as compared to neutral cues evoked higher activations in the medial prefrontal cortex, amygdala, and fronto-parietal regions, as also reported in prior studies [31–36, 38]. In contrast, bilateral PHG showed lower activation in response to drug vs. neutral cues in CU. Although not typically implicated in cue-reactivity studies in cocaine using populations, an earlier work showed that smoking as compared to neutral cues evoked “deactivation” of bilateral PHG in smokers relative to non-smokers [83]. The PHG appeared to show higher cue-elicited activation in link with the severity of dependence in drug users [84–87]. Diminished cue-related PHG activation was associated with better treatment retention in CU [88], whereas higher activity was associated with relapse in smokers undergoing cessation treatment [85]. In heroin users the PHG showed higher cue-reactivity [89], and methadone maintenance treatment reduced PHG cue response [90]. Thus, the finding of less cue-induced “deactivation” of the PHG in association with chronic cocaine craving is consistent with this literature. These findings also suggest the complexity of cue-elicited responses [86] and the importance in considering inter-subject variation in drug use variables and abstinence status to fully evaluate cue reactivity.

### LC functional connectivity in response to drug cues in cocaine users

Cue exposure frequently precedes drug seeking and consumption. Here, using a cue-reactivity paradigm, we demonstrated that both cue-elicited PHG activity and PHG-LC connectivity relate to chronic cocaine craving. Made possible by precisely localizing the LC with neuromelanin imaging, these findings provide direct evidence implicating the NA system in cocaine misuse. LC and PHG interaction may contribute to the retrieval of episodic memory [65, 78, 91–94] in relation to drug use. Earlier studies showed increased hippocampal and parahippocampal cue reactivities in relapsors vs. non-relapsors and as a predictor of relapse in drug users [95–97]. Consistent with this literature, the positive correlation of both cue-elicited PHG activity (less “deactivation”) and PHG-LC connectivity with craving supports the role of the NA circuit in the activation of drug-associated memory. Decreased suppression of the PHG may result in overall excitatory effects on the circuits of memory, interoception, reward/salience, and motivational drive [98] and contributes to drug use. Indeed, on the basis of causal modeling of the drug-cue processing neural network in cocaine users, an earlier study suggested a hippocampus→parahippocampal gyrus→orbital frontal cortex→ventral striatum pathway that associated reward/motivational processing with chronic cocaine use [99].

### Limitations of the study and conclusions

A number of issues need to be considered. First, LC connectivities support chronic cocaine craving by engaging the PHG memory system. Untoward in the context of cue exposures, this circuit may also be involved in adaptive processes central to a healthy cognition. A study combining both cue-reactivity and nondrug-related memory tasks will show how PHG LC circuit is involved in memory encoding and retrieval in CU. Second, LC is a small structure, and, although the great majority of neuromelanin images were acquired with 3-Tesla machines, there has been discussion whether a 7-Telsa magnet demonstrates higher sensitivity. A recent work concluded that, while there is insufficient evidence to prefer the 7-T SPIR sequence over the 3 T TSE sequence, the isotropic voxels at 7 T confer an advantage in visualizing the LC [100]. Third, as discussed earlier, LC NMS increases to a peak around 60 years of age and starts to decline afterwards [101], along with deterioration in functional compensation for the age-related changes. It remains to be seen whether aging of the LC circuits, including the connectivity with the PHG, may account for diminution in drug craving in CU [102].

In conclusion, we demonstrate for the first time the effects of cocaine misuse on LC NMS and LC circuit response during cue exposure. The findings suggest a critical role of the NA system in supporting craving and may facilitate more research of NA dysfunction in cocaine addiction.

## Funding and disclosure

Supported by NIH grants DA044749, DA023248, DA040032, and DA045743, as well as the Department of Mental Health and Addiction Services (DMHAS) of the State of Connecticut. The funding agencies otherwise have no roles in the conceptualization of the study, data collection and analysis, or the decision to publish these results. The authors declare no competing interests.

## Supplementary information

Supplement
