# Supplementary material for: Noradrenergic correlates of chronic cocaine craving: neuromelanin and functional brain imaging
Source: Neuropsychopharmacology. 2021 Jan 6;46(4):851–9. doi: 10.1038/s41386-020-00937-9 (PMC8027452; doi:10.1038/s41386-020-00937-9)
Supplement: Supplementary file 1 — Supplement [file 41386_2020_937_MOESM1_ESM.docx]

**Supplement**

**Wang et al., Noradrenergic correlates of chronic cocaine craving: neuromelanin and functional brain imaging**

**Supplementary Table S1.** Cue-elicited bilateral PHG connectivities (gPPI) in CU.

| Regions | Cluster | Voxel | MNI Coordinates (mm) | | |
| --- | --- | --- | --- | --- | --- |
|  | Size (# voxels) | Z Value | X | Y | Z |
| Bilateral PHG gPPI | | | | | |
| ventromedial prefrontal cortex | 1220 | 5.88 | 0 | 59 | -5 |
| R Caudate | (same cluster) | 5.06 | 9 | 8 | -2 |
| R middle temporal G | 1185 | 5.82 | 51 | -61 | 1 |
| L middle temporal G | 822 | 5.53 | -51 | -70 | 1 |
| R superior parietal lobule | 564 | 4.75 | 39 | -34 | 46 |
| L superior parietal lobule | 158 | 4.55 | -33 | -40 | 46 |
| L inferior frontal G | 432 | 4.41 | -45 | 38 | 4 |
| L Amygdala | 102 | 4.26 | -27 | 5 | -20 |
| R superior frontal G | 136 | 4.16 | 24 | -7 | 55 |
| L superior temporal G | 108 | 3.91 | -60 | -7 | -2 |
| L superior frontal G | 135 | 3.81 | -18 | 41 | 49 |

Voxel p<0.001, uncorrected, in combination with cluster p<0.05, FWE-corrected. L: left; R: right. G: gyrus.
